# Recent Purification Technologies and Human Health Risk Assessment of Microplastics

**DOI:** 10.3390/ma13225196

**Published:** 2020-11-17

**Authors:** Jun Woo Park, Su Jin Lee, Dae Youn Hwang, Sungbaek Seo

**Affiliations:** Department of Biomaterials Science (BK21 FOUR Program), College of Natural Resources and Life Science/Life and Industry Convergence Research Institute, Pusan National University, Miryang 50463, Korea; qcwalq@pusan.ac.kr (J.W.P.); nuit4510@pusan.ac.kr (S.J.L.)

**Keywords:** microplastics, purification, identification methods, risk assessment

## Abstract

Microplastic (MP)-based contaminants in the environment are pervasive, but standard technologies used for MP identification have not yet been reported. Human beings take up MPs from the environmental ecosystem through the food chain without any particular purification. MPs can penetrate into capillaries from the bloodstream, resulting in endocrine system disorders or toxicity. In this review, we introduced several technologies, such as filtration using membranes, biological degradation, electrocoagulation, and removal using nanoparticles, used for the purification of MPs or related contaminants. Current studies of identification methods of MPs and evaluation tests of MPs exposure-based harmfulness in vitro and in vivo were summarized.

## 1. Introduction

In recent years, plastic waste and pollution have become widespread in environmental ecosystems, causing harm to human health [1,2]. Plastic pollution ranges from oceanic [3] and terrestrial [4] to atmospheric [5] pollution. Plastics are broken down to microplastics (MPs), which are <5 mm in size. There are two types of MPs—primary MPs that are intentionally manufactured (detergent) and secondary MPs that are generated via mechanical collision, biodegradation, and photo-oxidative degradation of primary MPs. Both types of MPs carry toxic organic pollutants and heavy metals, since they have large surface areas and strong hydrophobicity. Furthermore, the toxic materials can be maintained for a long time due to the chemical stability of MPs for thousands of years. MPs are ingested and accumulated in plants/animals and finally humans via the food chain. Therefore, purification of MPs by removing and/or extracting is required. However, methods of identification, quantification, and exposure assessment evaluation of MPs have rarely been established.

For developing improved purification technologies to treat MPs, understanding principles of the technologies, their advantages, and limitations is essential. Since the mechanism of the purification is based on properties of plastic materials, we need be aware of the basic chemical/physical properties of MPs. Accordingly, in this review, we will introduce the origin of microplastics and types of microplastics. We reviewed current technologies used to purify MPs and current studies on the evaluation of the harmfulness of MPs in vitro and in vivo to determine potential human health risks. Several approaches, such as filtration using membranes [6,7,8], biological degradation by microorganisms [9,10], chemical coagulation and filtration [11,12,13,14], electrocoagulation [15], and extraction using photocatalytic micromotors [16] or magnetic nanoparticles (NPs) [17,18] used to purify MPs were introduced. The removal efficacy, size, and type of MPs used, and the advantages and limitations of the current techniques were tabulated. In vitro [19,20,21] and in vivo [22,23] impact assessment was introduced in view of current studies on MP identification or quantification [24,25].

## 2. Origin of Microplastics

**Figure 1:**
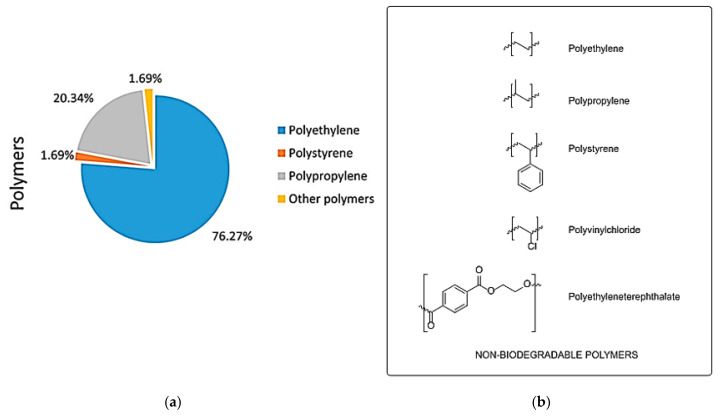
Microplastic abundance in the Pelagos Sanctuary (**a**) [34]. Chemical structures of non-biodegradable polymers (**b**) [35]. (Reprinted with permission from [35]. Copyright 2017 Elsev.).

The production of plastics has increased dramatically upon the growth of the plastic industry, and 420 million tonnes (MT) were produced in 2017 [1]. Wasted plastics will increase to 155–265 MT annually by 2060, most of which enter the world’s oceans through seawater or wastewater [26]. Plastics have been added with polymers to improve stability for various applications, and for this reason, the degradation of plastics takes a considerable amount of time [27,28]. Wasted plastics in the ocean are decreased in size due to prolonged external exposure (light, mechanical wear, waves, biodegradability, etc.) to form microplastics, or to intentionally form microplastics for products in industry [29,30]. Marine organisms eat the microplastics in the sea, and when humans eat these organisms, microplastics are exposed to humans [31]. In addition, microplastics were recently discovered in tap water and bottled water. In the case of tap water, 81% of particles with a size of 0.1 to 5 mm were found in 159 samples of tap water around the world, with 5.45 per liter [32]. In bottled water, 325 microplastics measuring 6.5 μm–5 mm were found per liter in 259 samples from 11 brands sold worldwide [33]. Microplastics are also found in commonly used bottled water or tap water, making them more easily exposed to humans. In the Pelagos Sanctuary, the microplastic is 76% polyethylene (PE), followed by polypropyrene (PP) and polystyrene (PS) [34] (Figure 1). PE, PP, and PS, which are the main components of microplastics, are non-biodegradable materials [35]. In addition, due to the characteristics of charge, hydrophilicity, and hydrophobicity, microplastics can easily combine with substances such as halogens such as bromine and toxic metals such as copper and lead to act as a carrier [36].

## 3. Current Technologies Used for MP Purification

### 3.1. Biological Degradation of MPs

Microorganisms can adapt to almost all environments, including those with plastic polymers. The microbes adhere onto the polymer surface for microbial colonization. Colonized microbes then excrete extracellular enzymes, resulting in hydrolytic cleavage of the polymer [37,38]. The polymer is degraded into polymers having low molecular weight and mineralized to carbon dioxide and water. Biological degradation of MPs using microorganisms is eco-friendly and environmentally safe for cleaning natural ecosystems.

**Figure 2:**
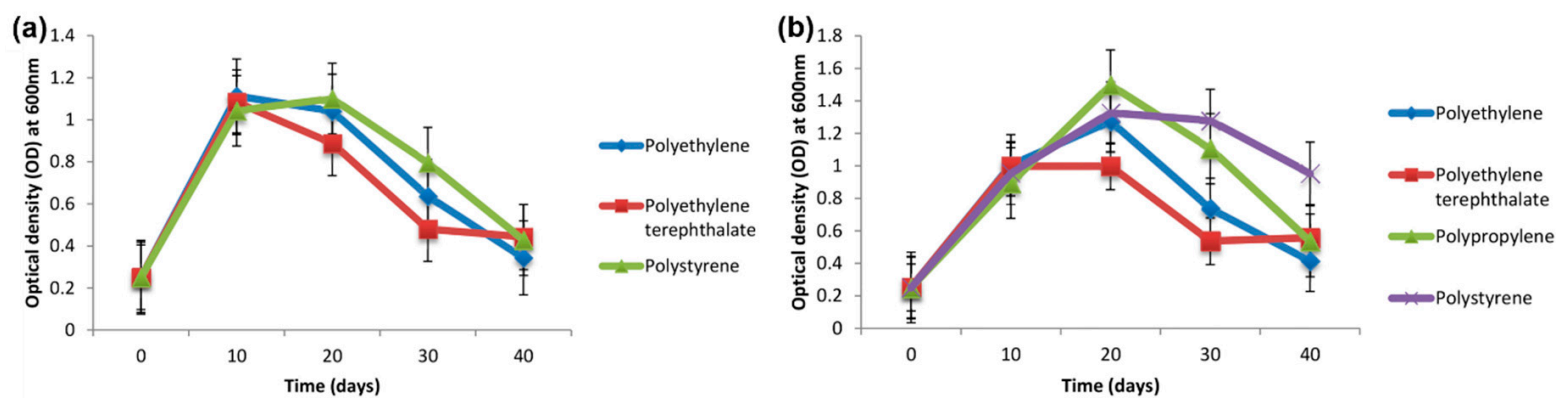
Growth curves of (**a**) *Bacillus cereus* and (**b**) *Bacillus gottheilii* used for microplastic biodegradation [10]. (Reprinted with permission from [10]. Copyright 2017 Elsev.).

Biodegradation of PE pellets by the marine fungus *Zalerion maritimum* has been previously studied [9]. The fungus reduced the mass and size of the micropellets and altered their molecular and chemical elements, as evaluated by attenuated total reflectance Fourier transform infrared spectroscopy (FTIR). Another study focused on the screening of bacterial isolates for the degradation of various MPs consisting of PE, polyethylene terephthalate (PET), PP, and PS [10] (Figure 2). Among the bacterial isolate candidates, two strains cultivated on a synthetic medium contain different types of MPs. The extent of biodegradation was evaluated by morphological and structural changes. After 40 days of incubation, the weight loss percentage (or removal efficiency) of PE, PET, and PS using one of the bacterial isolates (*Bacillus cereus*) was 1.6%, 6.6%, and 7.4%, respectively. The weight loss percentage of PE, PET, PP, and PS using another bacterial isolate (*Bacillus gottheilii*) was 6.2%, 3.0%, 3.6%, and 5.8%, respectively.

### 3.2. Coagulation

Coagulation and subsequent ultrafiltration is a notable approach for the removal of pollutants in water plants due to the outstanding purification quality of the effluent. Recently, coagulation used for MP removal has attracted interest. Both Fe- and Al-based salts have been commonly used as coagulants [39,40]. Coagulation-mediated flocculation contributes to MP trapping and/or sweeping. Al-based coagulants may cause potential side effects in humans; for instance, taking in residual aluminum in water has been thought to be neurotoxic [41]. The effect of the size and surface status of MPs is important for the efficacy of coagulation and further purification. The removal efficiency of pristine MPs using the coagulant at a very high dose (>60 mg aluminum/L) is <10%. This can be attributed to the weak interaction between the pristine plastic surface and the coagulant. This interaction is increased when real samples from the environment are used, since the MP surface is weathered due to photooxidation and fragmentation.

Electrocoagulation is used to prepare the coagulant for MP purification electrically using metal electrodes. This process is simple and robust. Metal ions, including Fe^2+^, Al^3+^, and OH^−^ ions, are generated via electrolysis of the electrodes. These ions then contribute to the production of metal hydroxide coagulants. The coagulants destabilize or break down the suspended particles or colloids, resulting in closer *van der Waals* forces. Additionally, the coagulant forms a sludge that traps the suspended solids. One research group attempted to remove PE beads from wastewater and studied the effect of pH, NaCl concentration, and current density in the wastewater environment. Removal efficiency of PE was >90% in pH ranging from 3 to 10 [15]. In this technical study, the removal efficiency of PE microbeads used as model MPs by charging neutralization via Al^3+^ ions and forming flocculation, followed by flotation or sedimentation, was >90% [15]. The effects of coagulation and flocculation on improving MP removal using PE and PS microspheres and polyester fibers were studied [42]. Therefore, electrocoagulation of MPs provides a cost-saving purification method that does not depend on chemicals or microorganisms.

**Figure 3:**
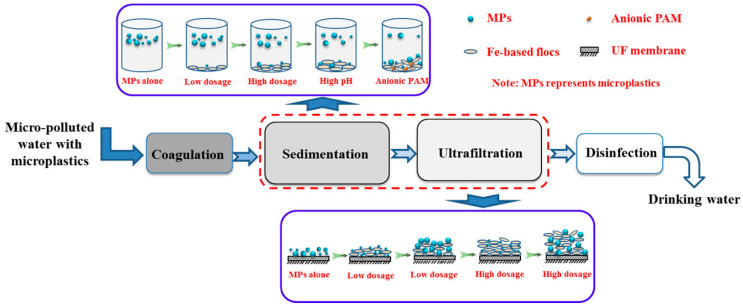
Schematic diagram of microplastics during the coagulation and ultrafiltration processes [43]. MP, microplastic; PAM, polyacrylamide; UF, ultrafiltration. (Reprinted with permission from [43]. Copyright 2017 Elsev.).

Monitoring the behavior of MPs during coagulation and ultrafiltration processes is required even in freshwater. In certain studies, the removal behavior of PE was investigated using chemical coagulants such as Al- or Fe-based salts and polyacrylamide (PAM) [43]. Figure 3 shows a schematic illustration of MPs during coagulation and ultrafiltration. Since individual MPs are suspended in water, coagulant-based floc formation allows better cake layer formation during the ultrafiltration process. By increasing the amount of coagulant, MPs can be efficiently filtered via the ultrafiltration process. Al-based salts removed PE MPs more efficiently than Fe-based salts. The further addition of anionic PAM contributed to the efficient removal of MPs due to the interaction with cationic Al-based flocs and the high adsorption capability of PAM. Unlike the non-treated PE MPs, the coagulated flocs were trapped in the ultrafiltration membrane efficiently. Under the optimized condition of addition of PAM at a high dosage, the removal efficiency of PE MPs was 90.9% [11].

**Figure 4:**
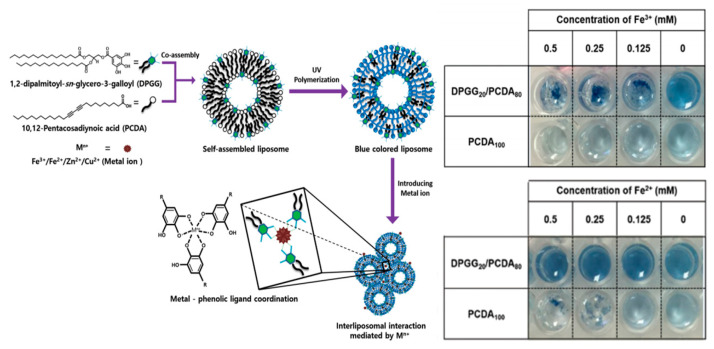
Schematic illustration of metal ion-mediated coagulation behavior of liposomes consisting of phenolic lipids. The coagulation was noticeable by increasing concentrations of Fe^3+^ ions [44].

In our studies, the coagulation of liposome-based particles (0.1~0.2 μm) was observed by introducing Fe-salt into surface phenolic liposome. Figure 4 shows the metal-phenolic coordinate bonds were attributed to trigger the coagulation. The Fe^3+^ concentration-dependent coagulation was clearly observed in aqueous solution. We believed that the chemical modification/incorporation of surface of MPs using phenolic molecules, then metal ion would be coagulants to generate coagulation of MPs. With coagulation of MPs, filtration efficiency to remove MPs would be enhanced for water treatment.

### 3.3. Filtration using Membranes

Membrane-based filtration is a commonly recognized technology used for water purification. Recently, membrane bioreactors have been connected with conventional membrane filtration technology to remove MPs from wastewater. However, this technique is not suitable for the volumes of water-based filtration because of low flow rates [17]. Furthermore, the removal efficiency of MPs using membranes particularly depends on membrane durability, influent flux, and MP size and concentration [45]. Filtration requires high pressure, energy, and cost. The removal efficiency of MPs via filtration technology using membranes is insufficient without membrane bioreactors [46].

**Figure 5:**
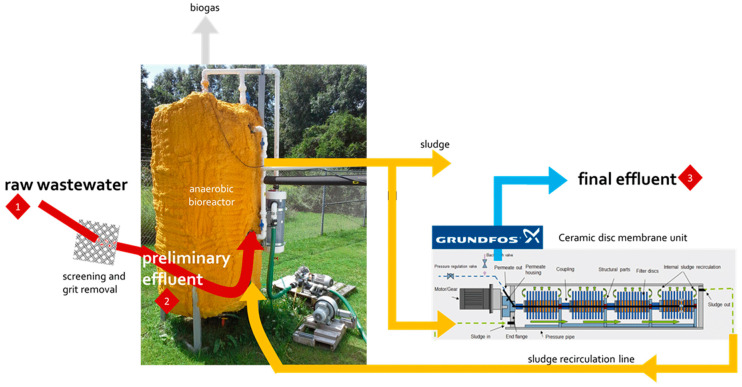
Schematic diagram of a pilot-scale anaerobic membrane bioreactor system equipped with three sampling zones: 1—raw wastewater, 2—preliminary effluent, and 3—final effluent [6].

The accumulation of MPs and small anthropogenic litter (SAL; e.g., cellulose products manufactured from the natural material) in aquatic environments is an increasing concern. To purify MP and SAL pollutants, wastewater treatment plants (WWTPs) have been developed (Figure 5). Purification is performed via several filtration-based treatments—activated sludge treatment as secondary treatment, granular sand filtration as tertiary treatment, and use of membrane bioreactor systems for microfiltration. The WWTP with secondary treatment removed 95.6% of the pollutants, the plant with tertiary treatment removed 97.2% of the pollutants, and the membrane bioreactor plant removed 99.4% of the pollutants [6]. However, MPs of size <20 μm are not retained or filtered in general WWTPs [47,48,49]. In summary, membrane-based filtration requires a complementary system for capturing smaller MPs without filter clogging [50].

### 3.4. Extraction of MPs Using NPs

Another water purification method is the extraction and removal of organic, inorganic, and microbial contaminants, including MPs, using NPs. A research group developed a polyoxometalate ionic liquid, which was adsorbed onto silica shell-magnetic core NPs, and studied the efficient and quantitative removal of water pollutants including MPs [17]. Another group suggested that hydrophobic Fe NPs, which could trap PE and PS beads (10–20 μm) and other types of MPs (>1 mm) at removal efficiencies of 92% and 93%, respectively, were recovered using a magnet [18]. In contrast to the traditional filtration technique, water purification using magnetic particles is suitable for the large volumes of water-based treatment without large infrastructure.

**Figure 6:**
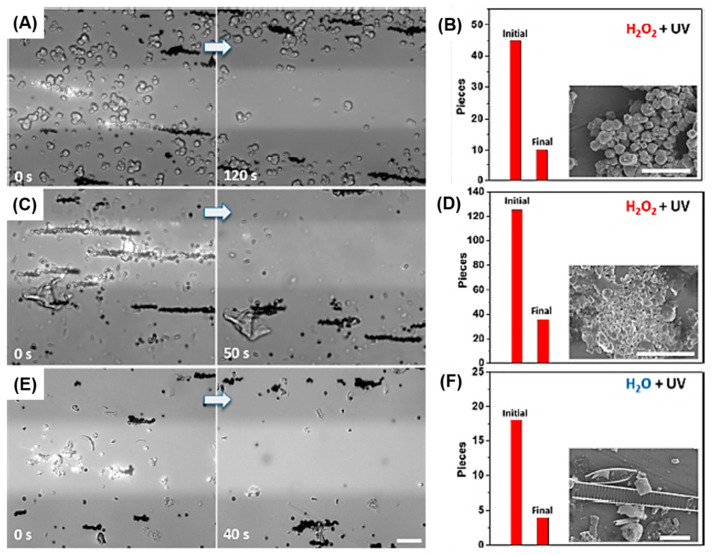
Captured images of the removal of different microplastics (MPs) using magnetic Au@Ni@TiO_2_ under a magnetic field: (**A**) washing powder sample in 0.1% H_2_O_2_ under 63 mW ultraviolet (UV) light; (**C**) face cleansing cream sample in 0.2% H_2_O_2_ under 63 mW UV light; (**E**) MP sample from the Warnow River in H_2_O under 315 mW UV light. (**B**–**F**) Amount of MPs in the initial (before removal) and final (after removal) stages is shown. The insets are scanning electron microscopy images. Scale bar, 10 μm [16]. (Reprinted with permission from [16]. Copyright 2019 American Chemical Society.)

One interesting strategy for MP removal is the use of self-propelled micromotors with a photocatalyst. A research group proposed TiO_2_ particle-based photocatalytic propulsion using a gold coating layer. In the presence of peroxide and water, the micromotor could travel efficiently under ultraviolet (UV) irradiation. The particles collected and removed MPs and suspended matter from environmental samples under real-time monitoring [16]. As shown in Figure 6, individual catalytic particles performed excellent collection efficiency and removal of suspended matter and MPs from environmental water samples. After 120 s, most of the zeolites from the washing powder were separated from the UV-illuminated area, resulting in a removal efficiency of approximately 77% (Figure 6A,B). MPs were extracted from a face cleansing cream sample in a 0.2% H_2_O_2_ solution. Similar to zeolites, MPs were removed with an efficiency of approximately 71% (Figure 6C,D). In only 40 s, 12 out of 18 MPs from a Warnow River sample were moved from the UV area using magnetic particles, reaching a removal efficiency of 67% (Figure 6E,F) [16].

Another approach for MP removal is the use of a metal-organic framework (MOF). MOFs show the benefits of high porosity, structure control, multiple functionalities, and charge for pollutant removal. One research group developed zirconium MOF-based foam as a platform for MP removal [51]. Under optimized conditions, a removal efficiency of ~95.5% was achieved. Additionally, MOF recycling and large-scale filtration could be performed.

In summary, Table 1 shows the different MP purification technologies and their experimental types, sizes, removal efficiencies, advantages, and limitations.

## 4. Current Technologies Used for MP Detection and Quantification Risk Assessment

### 4.1. MP Identification and Detection

Stereo microscopy is generally used for the identification of MPs of hundreds of micrometers in size. It is a simple, fast, and easy MP identification method. Since MPs show no color or typical shape, it is very difficult to distinguish them from natural source-based particles/fibers. SEM enables observation of highly magnified images, including information on the size and surface texture of MPs. Further energy-dispersive X-ray spectroscopy (EDS) allows the determination of the elemental composition and relative quantification of the objects. This information is helpful for distinguishing carbon-based MPs from inorganic particles. However, scanning electron microscopy (SEM)-EDS is expensive and requires more time for sample preparation and examination. The color of the MPs cannot be detected using this equipment.

The characteristic chemical bonds of carbon-based MPs can be easily identified using FTIR. The unique spectra of MPs discriminate them from other organic and inorganic particles and help in identifying the different types of MPs. Micro-FTIR (μ-FTIR) is used for the chemical identification and observation of microscopic images of micro-sized MPs. Identification and simultaneous quantification of fiber polymers can be performed by FTIR. To confirm the width and length of the fiber, SEM can also be used [55].

**Figure 7:**
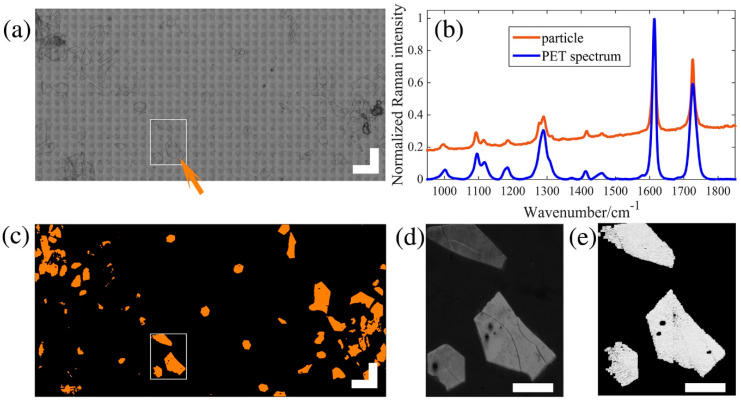
Stimulated Raman scattering (SRS) and Raman mapping of a nail polish extract. The tile scanned white light image of the measured area is shown in (**a**); scale bar: 500 μm. A particle (indicated by the orange arrow) in this image was measured with conventional Raman for confirmation, and its spectrum is shown in (**b**); orange spectrum, particle). The blue curve is a Raman reference spectrum of polyethylene terephthalate (PET) for comparison. In the SRS overlay image (**c**), five binary versions of the five identification images were color coded and overlaid as follows: PET: orange; Nylon: red; polystyrene: green; and polypropylene and polyethylene were not found; scale bar: 500 μm. (**d**) is a spontaneous Raman mapping from the area marked with a white square in (**a**) and (**c**), fitted with direct classical least squares to a reference spectrum of PET. (**e**) is the same area of the PET identified image with SRS, with greyscale values indicating the identification scores. Scale bars in (**d**,**e**): 200 μm [56].

According to the molecular structure and atoms in MPs, different frequencies of back-scattered light, which appear in the spectra of MPs, can be detected. Similar to FTIR, Raman analysis provides information on the characteristic spectra of MPs and the polymer composition of the sample. Raman and FTIR spectra used for identifying complex MPs support each other. MPs remain intact when the non-contact mode of Raman analysis, compared with FTIR, is used. However, Raman spectroscopy is sensitive to self-fluorescent pigments and additives in MPs, thereby interrupting the clear identification of MPs. An analytical method used for the characterization and quantitation of MPs of various sizes (63–600 μm) was analyzed by Raman microscopy. A research group proposed a fast identification method using Raman scattering microscopy (Figure 7). They identified 5 different types of MPs, among 88 MPs, from environmental samples and consumer products in <5 h by converting 12,000 particles per kg dry weight [56].

These methods characterize the specific thermal stability of MPs. DSC has been used to identify different types of polymers, including PE; however, it is difficult to distinguish PES from PET due to overlapping phase transition signals. Pyrolysis-gas chromatography-mass spectrometry (Pyro-GC-MS) is an analytical method, which uses thermally decomposed gas from MPs. A relatively small amount (0.35–7 mg) of MPs can be pyrolized at a high temperature (700 °C) and then analyzed using GC-MS. This thermal analysis is an alternative technique used for the chemical identification of MPs via spectroscopic analysis. However, the following steps in MP analysis cannot be performed due to the destructive method.

### 4.2. MP Quantification

Since MPs have aroused serious concern worldwide, analysis of MPs from environmental samples is required. Multiple processes of extraction, isolation/separation, identification, and quantification must be performed. Here, we focused on the MP identification and quantification methods. To identify and quantify MPs, several analytical methods such as optical/fluorescence microscopy [57], scanning electron microscopy (SEM), FTIR [58,59], Raman spectroscopy [60], thermogravimetric analysis [61], differential scanning calorimetry (DSC), and mass spectroscopy [62,63] have been used [24,64,65].

**Figure 8:**
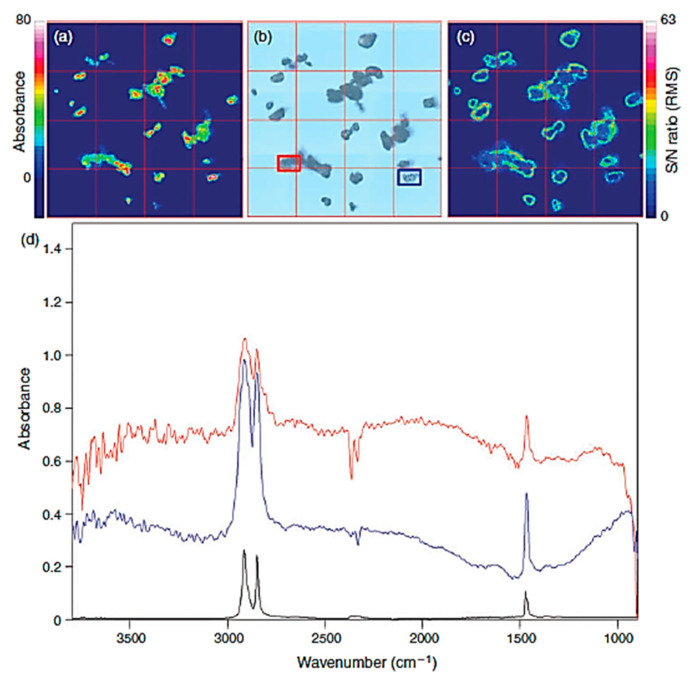
Results of transmittance focal plane array (FPA) detector-based micro-Fourier-transform infrared imaging of polyethylene (PE) powder on a blank CaF2 crystal. (**a**) Imaging of the wavenumber 2980–2780 cm^−1^. (**b**) Visual picture of the PE sample. (**c**) Signal-to-noise (S/N) ratio, effective noise calculated with the root mean squares (RMS) method, in the wavenumber range 2980-2780 cm^−1^. (**d**) Spectra acquired at a point of intermediate intensity (red spectrum, point marked by red square in (**b**)) and at a point of high intensity (blue spectrum, point marked by blue square in (**b**)), PE reference spectrum in black. The colour bars represent the intensity of the integrated band or S/N ratio. The edge length of a red outlined FPA field is 170 μm [69]. (Republished with permission of CSIRO, from ref. [69]; permission conveyed through Copyright Clearance Center, Inc., Danvers, MA, USA).

The most common method of quantifying microplastics is an optical microscope, which is calculated visually [66]. However, this method has many limitations in terms of accuracy, and it cannot distinguish plastics or quantify small sizes due to the resolution of the microscopy [67,68]. In order to overcome microscopy, other studies have performed quantification by combining microscope and spectroscopy (microscope and Fourier transform infrared spectroscopy (FTIR)) (Figure 8) [69].

Raman micro-spectroscopy is a technique that can identify microplastics through surface technology using Raman, and can quantify it by combining it with a microscopy [70,71]. A wide range of sizes can be identified and quantified, and particles smaller than 1 μm are possible [72]. Scanning Electron Microscopy (SEM) uses a high-energy focused electron beam to magnify a specific sample area. The analysis area evaluated by SEM can also be elementally analyzed using Energy Dispersion Spectroscopy (EDS). Microplastics can be identified using SEM and quantified by elemental analysis using EDS. SEM/EDS was used to confirm the identification and quantification of microplastics in sea trawl and fish intestines [73,74]. Reflectance micro FT-IR imaging based on focal plane array was used to identify and quantify microplastics in wastewater. The authors say that this method is useful for identifying and quantifying microplastics in wastewater [75].

Here is a summary table of MP identification methods modified from reference [24] in Table 2.

## 5. Current Technologies used for MP Risk Assessment

### 5.1. Toxicity of MPs/NPs in Human Cells

**Figure 9:**
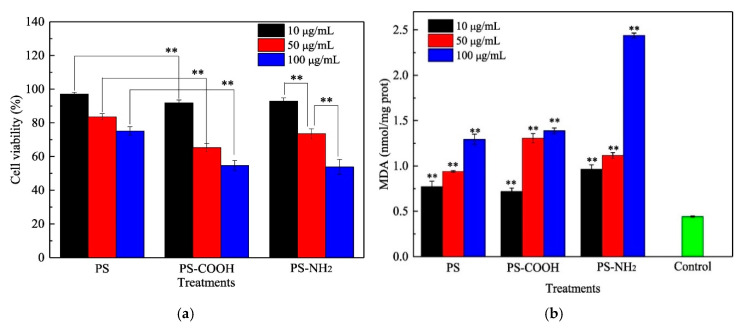
Effects of PS, PS-COOH, and PS-NH_2_ at different concentrations on cell viability and malondialdehyde (MDA) content in HepG2 cells. (**a**) The cytotoxicity of the NPs was measured in HepG2 cells using 3-(4,5-dimethylthiazol-2-yl)-2,5-diphenyltetrazolium bromide (MTT) viability assay. (**b**) The MDA level for oxidative stress in HepG2 cells after treatment of NPs was detected using the corresponding detection kit. Control indicates HepG2 cells treated with the vehicle. ** *p* < 0.05, according to the analysis of variance test [20]. (Reprinted with permission from [20]. Copyright 2017 Elsev.)

Recently, impact assessment of several MPs/NPs has been performed in various cells derived from human tissues. However, these assessments on the toxicity of MPs/NPs against human cells showed conflicting results. Most of the studies suggested that MPs/NPs induced some degree of toxicity or pathological changes in human cells, but a few studies showed that these MPs/NPs did not show any significant cellular toxicity, except at high concentrations. First, significant toxicity was detected in human cells treated with various MPs/NPs, including PS, carboxylated PS, PE, and PP. T98G and HeLa cells showed increased cytotoxicity after treatment with PE MPs (3–15 μm) or PS MPs (10 μm), and similar toxic effects were detected in Caco-2 and BEAS-2B cells treated with PS MPs (0.1–5 μm) [76,77,78]. Additionally, smaller PP particles (20 µm) induced some degree of toxicity at high concentrations in HDFs and Raw 264.7 cells, whereas larger PP particles (25–200 μm) did not induce toxicity [79]. Some small PS NPs (<100 nm) induced significant toxicity in THP-1, DMBM-2, and BEAS-2B cells at very high or low concentrations [80,81]. Furthermore, human HepG2 cells were treated with PS (50 nm) with three distinct surfaces (PS, PS-COOH, and PS-NH_2_) to assess the toxic effects. The viability of HepG2 cells treated with PS (10, 50, and 100 µg/mL) was remarkably decreased in a dose-dependent manner (Figure 9).

**Figure 10:**
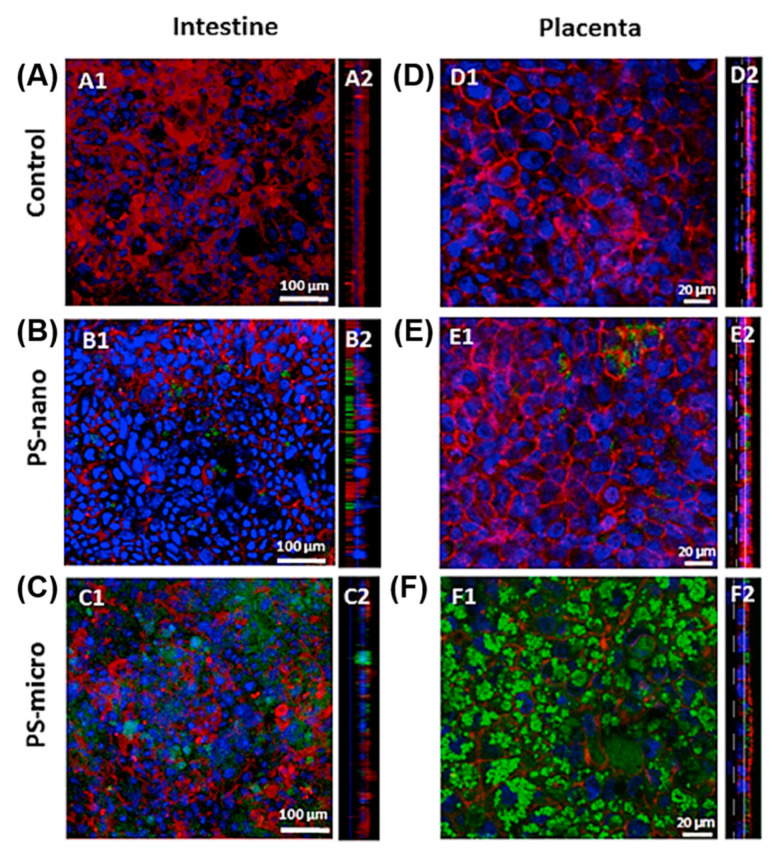
Internalization and intracellular distribution of PS NPs and PS MPs in intestinal and placental cocultures. Caco-2/HT29-MTX-E12 (**A**–**C**) and BeWo b30/HPEC-A2 (**D**–**F**) cocultures were observed by confocal microscopy after exposure to PS NPs (**B**,**E**) and PS MPs (**C**,**F**) (100 μg/mL) for 24 h. Cells were stained with phalloidin (actin, red) and Dapi (nuclei, blue), whereas PS particles (Rhodamine 6G) were fluorescently labeled (green). Also, A1–F1 show single z-plane of the cell layer, while A2–F2 showx-z-cross-section views [21]. (Reprinted with permission from [21]. Copyright 2017 Elsev.)

Meanwhile, significant pathological and physiological alterations were detected in MP/NP-treated human cells during the induction of cytotoxic effects. Oxidative stress was remarkably enhanced in PS-treated BEAS-2B cells, PE MPs-treated T98G and DMBM-2 cells, and PS NPs-treated Hs27 cells [76,78,82]. The secretion of inflammatory cytokines, including interleukin (IL)-6, IL-8, and tumor necrosis factor-α (TNF-α), was stimulated by treatment of carboxylated PS-nano in U937, THP-1, DMBM-2, and A549 cells and PP-micro in peripheral mononuclear blood cells [80,83]. Additionally, treatment with PS (50 nm) with three distinct surfaces (PS, PS-COOH, and PS-NH_2_) exhibited a decrease in superoxide dismutase (SOD) activity and an increase in the MDA and glutathione contents (Figure 10) [20]. Cells treated with some types of MPs showed alterations in the mitochondrial membrane potential, ABC transporter activity, histamine release, cell cycle arrest, apoptosis, autophagy, and endoplasmic reticulum stress response [77,81,83].

On the contrary, a few other studies have provided evidence that MPs/NPs cannot induce any significant cellular toxicity, even though they are normally taken up by the cells. PET NPs showed no apparent toxic effect in Caco-2 cells, but it was internalized into the endo-lysosomal compartment, thereby crossing the Caco-2 intestinal barrier [84]. Additionally, PS MPs (1, 4, and10 μm) did not affect the viability of Caco-2 cells and the THP-1 monocytic line, except at very high doses [85]. No significant toxicity of COOH-modified PS was observed in Caco-2/HT29-MTX-E12 co-culture and BeWo b30 cells; additionally, they were not evenly distributed in the layer of both cells after internalization (Figure 10) [21].

### 5.2. Toxicity of MPs/NPs in Mice and Rats

**Figure 11:**
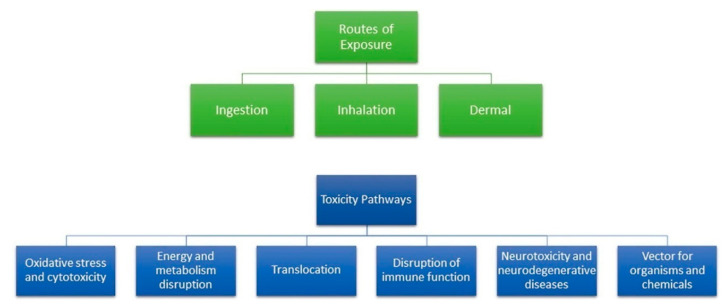
Three exposure routes and various toxicity pathways of MPs in the human body [87].

Recently, the toxicity of MPs/NPs in human and marine organisms has attracted attention because the utilization and environmental distribution of MPs/NPs has remarkably increased [86,87]. The effects of MPs on human health and physiology are little known; however, since MPs are well-known ubiquitous environmental contaminants, human exposure to MPs is inevitable. MPs can travel through the entire human body through various exposure routes including ingestion, inhalation, and dermal contact because they are distributed differently in products, foods, and air. After exposure to MPs, various toxicity pathways, including oxidative stress, neurotoxicity, and metabolism disruption, are activated, as shown in Figure 11 [88]. Due to these complexities in humans, animal studies must be performed to assess the risk of MPs/NPs on human health and physiology.

Until now, many studies have reported the toxicological and environmental effects of MPs on the physiology and behavior of marine organisms and ecological processes [89,90]. Most of these studies have focused on large marine vertebrates such as fish, showing that MPs/NPs could accumulate in the larval and adult gut, gills, and liver [91,92]. However, toxicity and accumulation of various types of MPs/NPs in experimental animals have been investigated in recent studies. However, these studies provide conflicting results on the toxicity and pathology of MPs/NPs in experimental animals, compared to that in human cells. Most of these studies suggested that MPs/NPs could induce various changes in toxicology and physiology, but a few other studies showed that they did not have any significant toxic effects in mice and rats. Furthermore, most studies only focused on PS of various sizes, and no other MPs made of different materials (Table 3). 

**Figure 12:**
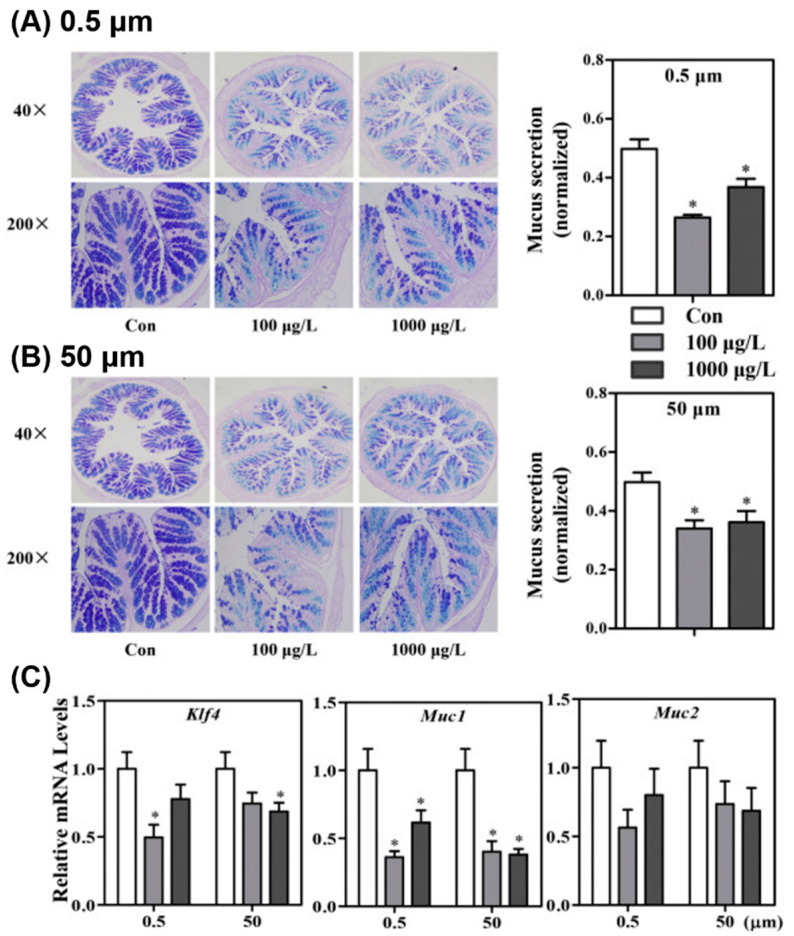
Effects of PS MPs exposure on mucus secretion in the gut. After alcian blue/periodic acid–Schiff (AB-PAS) staining, mucus secretion was normalized using the ratio of the mucus secretion area to the entire colon area. Institute of Cancer Research (ICR) mice were treated with (**A**) PS MPs (0.5 μm) and (**B**) PS MPs (50 μm) at two different concentrations. (**C**) The transcription levels of three genes related to mucin secretion in the colon are shown. The presented values are the means ± standard deviation (n = 8). * *p* < 0.05 versus control [22]. (Reprinted with permission from [22]. Copyright 2017 Elsev.)

First, PS MPs (5 and 20 μm) and a mixture of PS/PE/organophosphorus flame retardants of different sizes were found to accumulate in only three major tissues (gut, liver, and kidney) of mice, but there were some variations in the major accumulation sites [19,93,94,95]. Additionally, these animals showed various pathological changes in the gut, liver, and metabolism after ingestion of PS MP/NP. Treatment with PS MPs of various sizes (10–50 μm) induced a reduction in mucus secretion (Figure 12), barrier dysfunction, inflammation, and microbiota dysbiosis in the gut [22,95,96,97]. In particular, the effects of PS MPs (5 μm) on gut physiology, including mucus secretion, ion transporter expression, microbiota composition, and bile acid profile, were completely investigated in Institute of Cancer Research (ICR) mice [94].

Liver pathological changes induced by MP/NP treatment include enhancement in lipid accumulation, changes in lipid profile, increase in inflammation, and changes in lipid metabolism markers [19,93,94,95]. Additionally, treatment with these particles contributes to energy, glycolipid, and bile acid metabolism dysregulation, oxidative stress induction, and decreased acetylcholinesterase [93,94,95]. Alterations in the histopathology and serum markers of the liver tissue were observed after treatment with PS MPs (0.5 and 5 μm) [96,98]. Moreover, several immunological responses were altered in PS MPs-exposed animals. An increase in IL-1α cytokine secretion and a decrease in the number of Th17 and Treg cells, among CD4^+^ cells, were observed after treatment with PS NPs (10–150 µm) [97].

On the other hand, few studies have shown the opposite results of the toxicity of MP/NP treatment. PS MPs (1, 4, and 10 µm) treatment for 28 days did not induce any significant tissue damage or inflammatory responses in mice [24]. Additionally, PS NPs (25 and 50 nm) treatment for 5 weeks exhibited no significant body weight alterations, oxidative stress, behavioral changes, and abnormalities in Wistar rats [99].

Therefore, it should be noted that all studies were limited since they did not fully analyze the correlation between the biological responses of cells or animals and the physicochemical properties of MPs/NPs. Additional multi-dose studies and model trials are necessary to clarify the toxicity of MPs/NPs of various morphologies and sizes in human cells and animal models.

## 6. Conclusions

MPs are found in the ocean, atmosphere, and even drinking water and can easily be exposed to humans. Therefore, techniques for MP removal or purification are required, and it is necessary to establish an evaluation assessment of the harmfulness of MPs. Current studies of methodologies, including chemical, biological, and physical methods to remove or purify MPs from the environment, have been abridged. There are many ways to purify MPs, such as filtration, degradation, coagulation, and extraction using NPs, but only small volumes of MP-containing samples can be purified. If the advantages of the various methods presented in this review are combined, it will be possible to remove MPs from the actual environment effectively. Depending on the type of cell and the type, size, and concentration of the MPs, they may induce cytotoxicity in vitro. Depending on the type and size of MPs, they may induce cytotoxicity in vivo. However, toxicological and/or pathological effects vary widely. The harmfulness of MPs must be evaluated under various conditions in further studies. 

## Figures and Tables

**Table 1:** A summary of MP purification techniques.

MP Purification Technologies	MPs Used	Size	Removal Efficiency	Advantages	Limitations	Ref
Filtration	Wastewater, surface water	100–5000 µm	88.1%	Efficient mix of sorption-biological treatment processes Low maintenance costs Simple operation	Act as secondary MP sources Disability to treat small-sized MPs Sludge aggregation Mechanical devices	[7]
	wastewater	20 μm–4.75 mm	97.2%			[6]
	PES, PET, PA, PE, PP	<5 mm	99.3%			[8]
Biological degradation	PE	250–1000 μm	43%	Simplicity and safety for large-scale use Low operating costs Practically applicable in different environments Flexibility to handle a wide range of wastewater characteristics and flows	Aggregation of microbial assemblages on the surface Environmental conditions cannot be easily controlled Difficulty in the analysis of products on a large scale Lack of reproducibility Difficulty in finding the suitable microbial community	[9]
	PE, PP, PET, PS	75 μm	1.6–7.4%			[10]
Electro coagulation	PE	-	>90%	No chance of secondary pollution Suitable for the removal of smallest particles Sludge minimization Energy efficiency Cost-effectiveness Flexibility for automation	Repeated need for replacing the sacrificial anode, cathode passivation Non-usable in areas without electricity	[15]
Chemical coagulation	PE	<5 mm	<90.9%	Suitable for the removal of small microparticles Controllable operational conditions Use of simple mechanical devices	Addition of chemicals to media Non-usable for large MPs	[43]
	PE, PS	180 nm–125 μm	<13.6%			[52]
	PET, PE, PP, PAM	1–100 μm	40.5–54.5%			[53]
Extraction	PP, PE, PTFE, PET	5–100 µm	67–77%	No need for using oil Treatment of large volumes of water Can be used without further infrastructure High speed	No report on waste management Can only be used for extracting MPs from binary mixtures	[16]
	PS, PE, PET, PVC	10–5000 µm	93%			[18]
	PS, seawater	100–200 nm	95.5%			[54]

MP—microplastic; PES—polyethersulfone, PET—polyethylene terephthalate; PA—polyamide, PE—polyethylene; PP—polypropylene; PS—polystyrene; PAM—polyacrylamide; PTFE—polytetrafluoroethylene, PVC—polyvinyl chloride.

**Table 2:** Features, resolutions, and applications of currently studied MP identification methods. (Republished with permission of Royal Society of Chemistry, from ref. [24]; permission conveyed through Copyright Clearance Center, Inc.)

Identification Method	Feature	Resolution	Application
Microscopy	Simple, fast, and easy No chemical confirmation No polymer composition data	<100 µm	-
Microscopy (+ FTIR/Raman) ^a^	Plastic confirmation of subset samples Polymer composition of major or typical plastic types Possibility of missing small and transparent plastic particles	<1 µm	Major or typical plastic types
FTIR spectroscopy ^b^	No possibility of false positive data by chemical confirmation of all the plastic-like particles Reduction of false negative data Non-destructive analysis Automatic mapping (FPA-reflectance) Laborious work and time consuming for whole particle identification	<10 µm	Well-known spectra can identify microplastics and polymer types.
Raman spectroscopy ^b^	No possibility of false positive data by chemical confirmation of all the plastic-like particles Reduction of false negative data Non-destructive and non-contact analysis Interference by pigments	<1 µm	Using molecular structure and atoms, identify microplastics and polymer types.
Thermal analysis	Simultaneous analysis of polymer type and additive chemicals (pyro-GC/MS) A few polymer identification (DSC) Complex data (pyro-GC/MS)	<10 µm	PE, PP, PVC, PS, PA, PET and chlorinated or chlorosulfonated PE

^a^ FITR or Raman analysis of subgroup of plastic samples. ^b^ FTIR or Raman analysis of whole particles.

**Table 3:** The toxicological, pathological, and behavioral changes in mice and rats on MP/NP treatment. (We modified a summary table referred from [23].)

Classification	Size	Accumulated Tissue	Toxicological, Pathological, and Behavioral Changes	References
Detection of significant toxicological and pathological changes				
PS	5 and 20 μm	Gut, liver, and kidney	Induction of inflammatory response and lipid accumulation in the liver Alteration in the lipid profile and impairment of energy metabolism (reduction in adenosine triphosphate (ATP) levels) Increase in liver oxidative stress markers and decrease in acetylcholinesterase activity	[19]
PS	0.5 and 50 μm	-	Decrease in body, liver, and lipid weights Decrease in mucus secretion in the gut Alteration in the gut microbiota Alteration in the hepatic lipid profile and expression of some genes related to lipid metabolism	[22]
PS and PE + OPFRs ^a^	0.5–1.0 μm	Gut and liver	Enhancement in OPFR-induced oxidative stress, neurotoxicity, and metabolic disorder	[93]
PS	5 μm	Gut	Dysfunction of the intestinal barrier Dysbiosis of the gut microbiota Induction of bile acid metabolic disorder	[94]
PS	5 and 20 μm	Gut, liver, and kidney	Toxicokinetic/toxicodynamic modeling of organ-bioaccumulation and biomarker responses Alteration in the oxidative stress, energy, and lipid metabolism markers	[95]
PS	0.5 and 5 μm	-	Alteration in serum and liver metabolic markers Induction of fatty acid metabolic disorder in the F1 offspring after exposure to maternal MPs	[96]
PS	10–150 μm	-	Alteration in the composition and diversity of gut microbiota Increase in the IL-1α secretion in the serum and decrease in the Th17 and T_reg_ cells, among CD^4+^ cells Induction of the inflammatory response in the small intestine after treatment with high-concentration MPs	[97]
PS	5 μm	-	Alteration in histopathology, and serum and hepatic markers for liver toxicity Alteration in the transcription of genes related to glycolipid metabolism Dysbiosis of the gut microbiota and dysfunction of the gut barrier Induction of intergenerational effects and long-term metabolic consequences in the F1 and F2 generations after exposure to maternal MPs	[98]
No detection of significant toxicological and pathological changes				
PS	0.025 and 0.05 μm	-	No significant change in the neurobehavioral consequences	[23]
PS	1, 4 and 10 μm	-	No significant change in body/organ weight and histopathological structure No significant change in the inflammatory response and oxidative stress Intestinal tissue uptake of a low number of particles	[55]

^a^ OPFR—organophosphorus flame retardant.
